# Twenty-Five Years of No-Touch Saphenous Vein Harvesting for Coronary Artery Bypass Grafting: Structural Observations and Impact on Graft Performance

**DOI:** 10.21470/1678-9741-2019-0238

**Published:** 2020

**Authors:** Ninos Samano, Domingos Souza, Bruno Botelho Pinheiro, Tomislav Kopjar, Michael Dashwood

**Affiliations:** 1 Department of Cardiothoracic and Vascular Surgery and University Health Care Research Center, Faculty of Medicine and Health, Örebro University, Örebro, Sweden.; 2 Department of Cardiothoracic and Vascular Surgery, Faculty of Medicine and Health, Örebro University, Örebro, Sweden.; 3 Department Cardiovascular Surgery, Hospital do Coração Anis Rassi, Goiânia, GO, Brazil.; 4 Department of Cardiac Surgery, University of Zagreb School of Medicine and University Hospital Centre Zagreb, Zagreb, Croatia.; 5 Surgical and Interventional Sciences, Royal Free Hospital Campus, University College London Medical School, London, UK.

**Keywords:** Coronary Artery Bypass, Guidelines, Myocardial Revascularization, Saphenous Vein, Mammary Arteries, Cardiology

## Abstract

The saphenous vein is the most common conduit used in coronary artery bypass grafting (CABG) yet its failure rate is higher compared to arterial grafts. An improvement in saphenous vein graft performance is therefore a major priority in CABG. No-touch harvesting of the saphenous vein is one of the few interventions that has shown improved patency rates, comparable to that of the left internal thoracic artery. After more than two decades of no-touch research, this technique is now recognized as a Class IIa recommendation in the 2018 European Society of Cardiology and the European Association for Cardio-Thoracic Surgery guidelines on myocardial revascularization. In this review, we describe the structural alterations that occur in conventional versus no-touch saphenous vein grafts and how these changes affect graft patency. In addition, we discuss various strategies aimed at repairing saphenous vein grafts prepared at conventional CABG.

**Table:** 

Abbreviations, acronyms & symbols				
ADRF	= Adipocyte-derived relaxing factor		NICE	= National Institute for Health and Care Excellence
ADV	= Adventitia		NO	= Nitric oxide
C	= Conventional		NT	= No-touch
CABG	= Coronary artery bypass grafting		NYHA	= New York Heart Association ok
CI	= Confidence interval		OVH	= Open vein harvesting
EACTS	= European Association for Cardio-Thoracic Surgery		PVAT	= Perivascular adipose tissue
ESC	= European Society of Cardiology		PVF	= Perivascular fat
EVH	= Endoscopic vein harvesting		RA	= Radial artery
ITA	= Internal thoracic artery		SV	= Saphenous vein
L	= Lumen		SVG	= Saphenous vein graft
LITA	= Left internal thoracic artery		TM	= Thick media
M	= Media		VSMCs	= Vascular smooth muscle cells

## SAPHENOUS VEIN AS A BYPASS CONDUIT

In a recent History of Medicine Perspective published in the New England Journal of Medicine, Jones DS^[1]^ outlines the important contribution to coronary artery bypass grafting (CABG) made by Rene Favaloro, who first introduced the saphenous vein (SV) as a conduit for coronary revascularization^[2]^. In the subsequent 50 years, the SV has become the most commonly used graft in patients undergoing CABG^[3,4]^, yet its failure rate is greater than the one from the left internal thoracic artery (LITA)^[5-7]^. According to some, it is also inferior to that from the radial artery (RA)^[8-10]^. Apart from the structural differences between arteries and veins, it is noteworthy that, in general, LITA and RA are harvested in such a way that the pedicle of the surrounding tissue remains intact. When harvesting the saphenous vein graft (SVG), Favaloro’s original instructions are followed: “Care must be taken to dissect only the vein, avoiding as much as possible the adventitia that surrounds it”^[11]^. This method of preparing the SV has become the conventional (C) approach used in most cardiac centres when carrying out CABG. Considerable vascular damage is inflicted when harvesting in such a way that the damage affects vein graft quality and patency. Consequently, affecting clinical prognosis in terms of reoperation rates and long-term survival^[12]^.

Over two decades ago, the ‘no-touch’ (NT) technique of harvesting SV was introduced^[13]^. Using an atraumatic method, the vein is removed with a pedicle of surrounding tissue and with minimal vascular damage^[14]^. Subsequently, a randomized trial comparing C SVG and NT SVG has shown that the latter is superior in terms of patency rates and left ventricular ejection fraction at 1.5, 8.5, and 16 years postoperatively^[15-18]^, as seen in Table 1 from Samano et al.^[17]^, 2015. Furthermore, NT SVGs exhibit a patency comparable to that of LITA at up to 16 years^[16,17,19]^, as seen in Figure 1 from Samano et al.^[17]^, 2015.

**Table 1:** Ratio of number of patent grafts to total number of grafts for the two surgical techniques at three follow-up time points (Samano et al.^[17]^, 2015).

	Conventional (C)			No-touch (NT)			Group difference in % patency*		
Follow-up (years)	1.5	8.5	16	1.5	8.5	16	1.5	8.5	16
No. of patients	46	37	27	45	37	27			
Single grafts	96/107 (90%)	68/87 (78%)	41/63 (65%)	103/109 (94%)	78/87 (90%)	55/67 (82%)	0.23	0.05	0.06
Sequential grafts	16/20 (80%)	10/14 (71%)	5/9 (56%)	15/15 (100%)	14/14 (100%)	7/8 (87%)	0.12	0.10	0.29
All grafts	112/127 (89%)	78/101 (77%)	46/72 (64%)	118/124 (95%)	92/101 (91%)	62/75 (83%)	0.08	0.01	0.03

* Tested with multilevel logistic regression, except for sequential grafts, for which Fisher's exact test had to be used because of small numbers and cells with no occluded grafts.

**Fig. 1:**
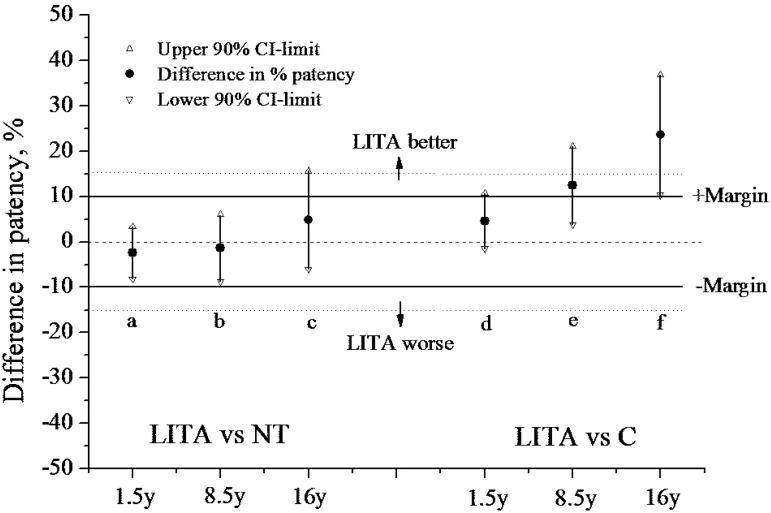
Differences in patency, left internal thoracic artery (LITA) vs. no-touch (NT) saphenous vein (SV) and LITA vs. conventional (C) SV at 1.5, 8.5, and 16 postoperative years. The six confidence intervals (a-f ) and the margins of 10 and 15 percentage units are the basis for comparing LITA with the SV with respect to potential equivalence and non-inferiority (Samano et al., 2015)^[17]^. CI=confidence interval.

Clinical outcomes have also been evaluated. There were significantly more patients free from angina and in New York Heart Association (NYHA) class I in the NT group *vs*. the C group at 8.5 years postoperatively^[20]^.

These higher patency rates and subsequent clinical advantages have led to the addition of the NT SVG harvesting technique as a Class IIa recommendation in the 2018 European Society of Cardiology (ESC) and the European Association for Cardio-Thoracic Surgery (EACTS) guidelines on myocardial revascularization^[21]^. Many cardiac surgeons in Sweden and Brazil as well as in other countries, including Korea, Japan, Croatia, China, and Russia, now use NT SVGs routinely.

## SAPHENOUS VEIN STRUCTURE AND VASCULAR DAMAGE

Here we provide an overview of the various aspects of vascular damage that occurs to the SV when using C *vs*. NT harvesting techniques and we discuss how this may affect the performance of such grafts used in patients undergoing CABG.

The damage to C compared to NT SVGs is obvious on visual examination, as seen in Figure 2 from Kopjar et al.^[22]^, 2016. While the surrounding cushion of fat remains intact in NT SVs, it is completely removed in C SVs. In addition, much of the vein’s outermost layer, the adventitia, is also removed or damaged. In C SVs, where SV has been distended at high intraluminal pressure (to overcome spasm), the tunica media is thinner than in non-distended NT SVs. The lumen of NT SV exhibits luminal folding as distension is not required, whereas the lumens of C SVGs are dilated, following high pressure distension. At the microscopic and ultrastructural level, tissue-specific damage and cellular alterations become more obvious, changes that will impact on graft quality and performance. These trauma-induced effects are seen in all three layers (tunica) of SV, the intima, media, and adventitia, as well as the surrounding cushion of perivascular fat.

**Fig. 2:**
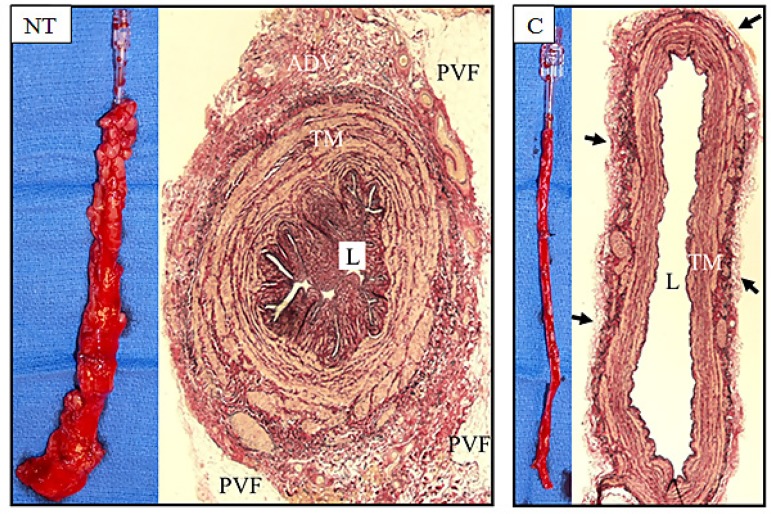
Examples of no-touch (NT) and conventional (C) saphenous vein (SV) grafts. The left panels show representative SV explants using both harvesting techniques. NT SV has its surrounding cushion of fat intact and has not been distended. C SV has the fat removed and has been distended to overcome spasm. The transverse section of NT SV shows an intact surrounding cushion of perivascular fat (PVF), an undamaged adventitia (ADV) and thick media (TM). As this vessel has not been distended, the lumen (L) is thrown into folds. The section of C SV exhibits various forms of damage. Much of the ADV has been stripped off, almost to the level of the external elastic lamina (small arrows). The media is thinner than that of the NT SV and L is grossly dilated, both due to high pressure intraluminal distension (Kopjar et al., 2016)^[22]^.

### Endothelium

This innermost cell layer lining, the intima, is virtually undamaged in NT SVs. This has been demonstrated by immunohistochemistry using endothelium-specific antibodies, a technique that allows quantitative assessment to be performed. In this way, a comparison of the NT SV *vs*. C SV’s endothelial integrity has been reported. Dramatic regions of endothelial denudation were observed in C SVs, resulting in an overall reduction in endothelial integrity^[23]^. This observation was confirmed by assessing the protein expression of CD31, a marker for endothelial cells^[24]^. At the ultrastructural level, striking shape changes of various cells in C SVs *vs*. NT SVs have been observed^[25]^, as seen in Figure 3 from Ahmed et al.^[25]^, 2004. For example, endothelial cells of NT SVGs remain intact with the nuclei protruding to the lumen and with junctions present between cells whereas those of C SVGs exhibit striking changes, including polymorphism of the endothelium, cells with ‘dark’ cytoplasm or very thin cell processes protruding towards the vein lumen. Fragments of squamous endothelial cells were abundant in electron-transparent cytoplasmic vesicles. Similar dramatic changes in the appearance of endothelial cells were also observed between NT SVs and C SVs when using scanning electron microscopy^[26]^.

**Fig. 3:**
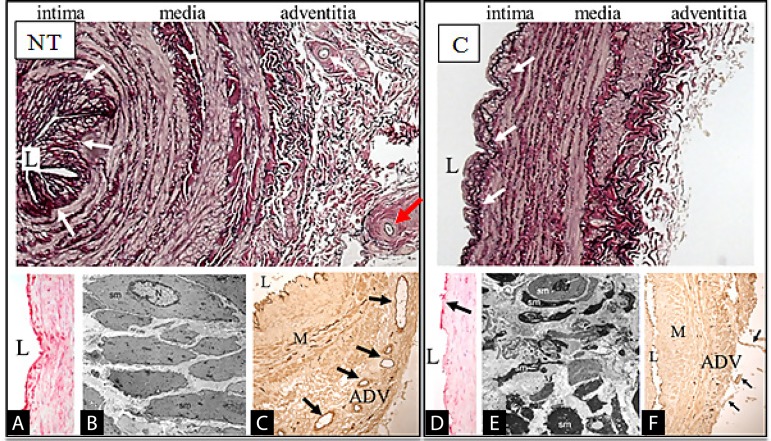
Histological, cellular, and ultrastructural comparison of notouch (NT) vs. conventional (C) saphenous vein (SV) grafts. Top panels are representative examples of part transverse sections through the wall of NT and C SVs stained with Elastic van Gieson and prepared for coronary artery bypass grafting. The intima surrounding lumen (L) of NT SV is thrown into folds. The vein ‘wall’ is thick with smooth muscle cells of the media (M) separated by the internal and external elastic laminae and with an intact adventitia (ADV) in which the vasa vasorum is located (red arrow). The intimal folds are absent in C SV, the vessel wall is thinner than that from NT SV (due to distension), and the ADV is mostly removed and damaged. The white arrows near vein L indicate the internal elastic lamina. The lower panels show damage caused to C SV when compared to NT SV. Panel A shows an intact endothelial lining of the NT vein L when compared to the C vein shown in panel D. Only a proportion of the endothelial cells in D stain red using the antibody CD31 (arrow). Panel B is a transmission electron micrograph showing the uniform shape and distribution of vascular smooth muscle cells in the M of a NT SV. The appearance of smooth muscle cells in the M of C SV is very different. Panel C shows the wall of a NT SV with an intact vasa vasorum (arrows) within the ADV. The ADV of C SV (F) is mostly removed with remnants indicated by the arrows. Endothelial cells of L and vasa vasorum stain dark brown in these sections with an intact layer lining the NT vein, but regions of denudation present in the C vein. The endothelial cells of the vasa vasorum at the M/ADV border are evident in panel C, but absent in panel F (Ahmed et al., 2004)^[25]^.

### Tunica Media

Vascular smooth muscle cells (VSMCs) in the media of NT SVs were of a regular arrangement, displaying normal and uniform morphological features and structured appearance, as seen in Figure 3. However, VSMCs in C SVs exhibited polymorphism and the presence of ovoid, elongated, or multi-shaped cells. In addition, the sarcoplasm of some cells was dark^[25]^. Similar ultrastructural observations were reported a decade later^[27]^. Interspersed within the VSMCs of the media is the vasa vasorum, a microvascular network that extends from the adventitia and provides this layer with oxygen and nutrients^[28,29]^. While the vasa vasorum of NT SVs remain intact and patent, those in C SVs exhibited morphological changes and were often occluded by plugging of erythrocytes^[25]^.

### Tunica Adventitia

While this outermost layer of SV is undamaged in NT SVs and the vasa vasorum remains intact, C SVs exhibit obvious signs of damage caused at harvesting and by manipulation by surgical instruments or when using endoscopic vein harvesting (EVH). In addition, the vasa vasorum of C SVs are disrupted, severing the connections to the media and potentially to the SV lumen^[26,28-30]^.

### Perivascular Fat

The pronounced surrounding cushion of perivascular fat that remains intact on NT SVs is completely removed when C SVs are harvested in traditional CABG. This perivascular adipose tissue (PVAT), is comprised mainly of discrete adipocytes that contain a network of capillaries and nerve fibres as well as a variety of other cell types, including macrophages, adipocyte stem/progenitor cells, lymphocytes, and fibroblasts. These cell types are suggested to possess various beneficial properties that may impact on blood vessel structure and function^[31,32]^.

## POTENTIAL CONSEQUENCES OF VASCULAR DAMAGE

### Endothelium

Damage to each of the layers of the SV may impact on its subsequent performance as a graft. The existence of a variety of endothelium-derived vasoactive factors have been recognised for many years. Of particular relevance to conduits used for revascularisation is nitric oxide (NO), a gaseous, endothelium-derived vasodilator that also possesses antiproliferative, antithrombotic, and anti-inflammatory properties^[33]^. Each of these properties is beneficial to graft performance and therefore damage to the endothelium will have adverse effects on the performance of the SVG used for CABG. Regarding the endothelial damage to C SVGs, the reduction in luminal NO levels would be expected to contribute to the spasm encountered during harvesting. This spasm is caused by surgical trauma when stripping the PVAT. In addition, platelet aggregation and thrombus formation will occur due to reduced local NO at regions of endothelial denudation and exposure of the intima basement membrane, factors involved in early graft failure. Apart from the obvious reduction in wall thickness of C compared with NT SVs, the ultrastructural alterations are striking under transmission electron microscopy^[25]^. The damage to the endothelium of C SVGs may be implicated in various aspects of graft failure, including an effect on intimal/neointimal development, local levels of endothelium-derived vasoactive agents, and platelet activity.

### Vascular Smooth Muscle Cells

The shape changes in VSMCs in C *vs*. NT SVs have been confirmed more recently, with evidence that that the molecular fingerprint of VSMC activation is primed in C compared to NT SVs^[27]^. Apart from affecting the reactivity, and therefore flow through SVGs, there is the potential for damaged VSMCs to undergo a phenotype change from ‘contractile’ to ‘synthetic’. This process is involved in neointimal hyperplasia, atheroma formation, and eventual graft occlusion.

### Adventitia

Whereas the adventitia of NT SVs remains intact, in C SVs this layer is severely damaged or almost completely removed. This procedure not only ‘weakens’ the vessel wall, making it more prone to the effect of altered haemodynamics once subjected to coronary arterial conditions, but also damages the vasa vasorum, a situation affecting transmural blood flow^[29]^. It is noteworthy that the vasa vasorum of veins is more pronounced and penetrates deeper into the media than in arteries where the circulating luminal blood supplies oxygen and nutrients. Experimentally, it has been shown that occlusion of adventitial vasa vasorum using a close-fitting external collar reduces or prevents transmural blood flow. This promotes neointimal hyperplasia and atheroma formation^[34]^. Furthermore, neointimal hyperplasia occurs if the adventitia is removed, but regresses on the appearance of ‘neoadventitia’^[35]^. These observations suggest that removal of the adventitia and damage to the vasa vasorum in C SVGs are involved in the poor patency rates reported for these veins when compared to the arterial grafts, mainly LITA. However, when using the NT technique, the adventitia and vasa vasorum remain intact and transmural flow is maintained. This has been confirmed where NT SVGs exhibit retrograde filling of the superficial adventitial vasa vasorum after implantation and removal of vascular clamps as well as when isolated segments are perfused with blood via the lumen^[29]^. Apart from histological evidence of damage to the adventitial vasa vasorum, ultrastructural observations show shape changes and plugging of erythrocytes in many of the remaining vasa vasorum, mainly within the media^[25,29]^. There is also the possibility that endothelial cells of the vasa vasorum may be involved in endothelial cell migration and subsequent re-endothelialization of regions of the SVG lumen affected at harvesting^[29]^.

### Perivascular Fat

The cushion of surrounding fat that remains intact in NT SVGs may have a number of beneficial effects. Firstly, this cushion possesses a mechanical/supporting role where it acts as a buffer and protects the vein from arterial haemodynamics once implanted into the coronary arterial circulation. In addition, this cushion helps prevent kinking of grafts of excessive length^[36]^. Furthermore, PVAT represents a source of so-called adipocyte-derived relaxing factor(s) (ADRF), that have potent relaxant or anticontractile properties^[31,32]^. Apart from these actions being demonstrated in blood vessels from experimental animals^[37-39]^, PVAT has been shown to possess anticontractile actions in isolated preparations of the human internal thoracic artery (ITA)^[40]^ and SV^[41]^. Although specific ADRFs have not yet been identified, various candidates have been suggested, including NO^[42]^, leptin^[32,43]^, H2S^[44]^, adiponectin^[45,46]^, and prostaglandins^[41,47]^. Clearly, the removal of such a layer with mechanical/buffering properties as well as a source of factors potentially beneficial to improved graft performance is likely to be inadvisable. It seems that Favaloro’s original instructions (1969) “…. to dissect only the vein…” may in some way account for the poor performance of C SVGs in CABG. The question arises; while arterial conduits (ITA and RA) are generally removed with a pedicle intact, why should the SV be harvested with the pedicle removed?

## MECHANICAL PROPERTIES OF PERIVASCULAR FAT: WHY USE UNNATURAL EXTERNAL SUPPORT?

The use of external venous supports in CABG is a strategy introduced over 50 years ago^[48]^. Various forms of external support have been described, ranging from fibrin glue^[49]^ to Dacron^TM((^^50,51]^ and external mesh^[52-54]^. More recently, an external support made from braided cobalt-chromium-nickel-molybdenum-iron alloy fibers^[55-57]^ has been tested. Based on the results from animal studies, a recent review discusses the proposed mechanisms of action of external SV supports in CABG patients^[58]^. For example, studies into the effect of external synthetic stents and sheaths in a pig model of vein into artery interposition grafting suggest that this form of support has a profound effect on vein graft remodelling and thickening. In addition, external stents inhibit neointima formation and reduce graft thickening via the promotion of angiogenesis in the space between the graft and the sheath or stent^[59]^. Disappointingly, when the results of the ‘EXTENT’ randomized trial on a small number of CABG patients were analysed, all ‘EXTENTED’ grafts were thrombosed whereas all LIMA grafts and non-stented SVGs remained patent^[51]^. The most recent encouraging data for external vein supports describes the use of braided durable, kink-resistant ‘VEST’ external stents made of plastically deformable cobalt-chrome wires^[58]^. Here, the placement of VESTs resulted in a significant decrease in mean intimal-medial area with a small decrease in intimal thickness between the stented and control groups. However, these were short-term follow-up studies in small patient numbers.

The main role of the external supports appears to affect mechanisms recognized to play an important role in vein graft performance. Such processes include altered shear stress, VSCM migration, intimal hyperplasia, and atherosclerotic plaque formation. While these processes are involved in graft failure when C SVGs have been used, they are reduced or abolished in vein grafts prepared by the NT technique.

A number of mechanisms have been identified that are suggested to explain the superior performance of SVs harvested by the NT technique^[60]^. Most mechanisms are related to preservation of the outer cushion of fat of the SV acting as ‘natural support’ and therefore sharing the same beneficial properties as those proposed for the various external stents described in the recent review by Mawhinney, Mounsey, and Taggart^[58]^, as seen in Figure 4 from Rueda et al., 2008^[36]^, and Mawhinney et al., 2017^[58]^. The ‘protective’ properties of the pedicle that is preserved on NT SVGs range from its ability to prevent kinking of long grafts^[36,61]^ and protection of the endothelium^[23,24]^ to the beneficial actions of perivascular fat-derived factors^[62,63]^ and preservation of an intact vasa vasorum^[29]^. Why then use synthetic external supports that may potentially be harmful, complicated, time-consuming to fit, or costly? It seems more logical to remove SV with a ‘natural’ perivascular support intact using the NT technique.

**Fig. 4:**
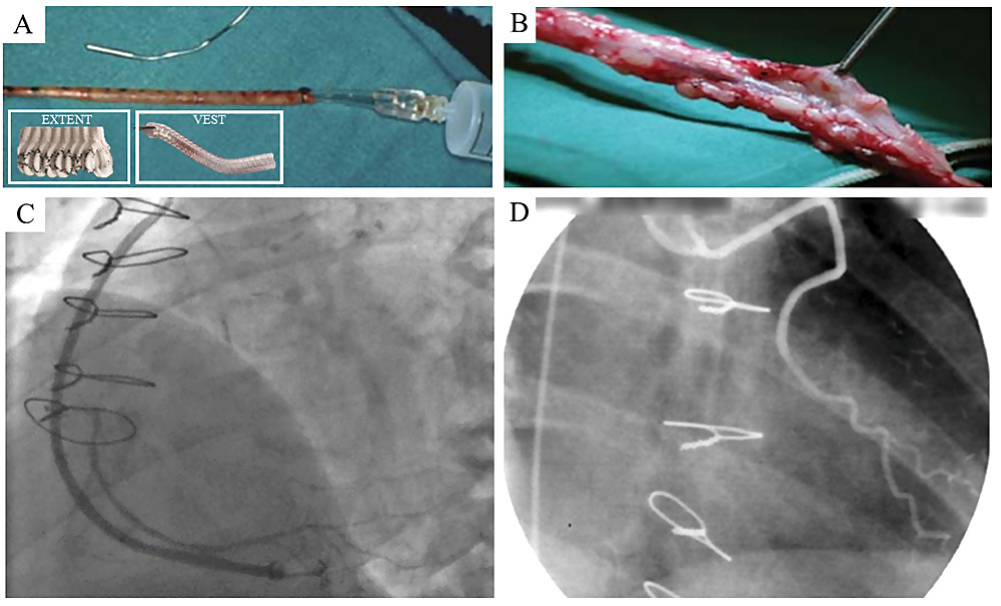
Unsupported and supported saphenous vein (SV) grafts for coronary artery bypass grafting. A) An example of a conventionally harvested SV with outermost tissue removed (Rueda et al.^[36]^, 2008). Insert, examples of DacronTM ‘EXTENT’ and ‘VEST’ external supports (Mawhinney et al.^[58]^, 2017). B) An example of a no-touch (NT) harvested SV with external tissue intact (Rueda et al.^[36]^, 2008). C) Angiogram of an SV with VEST at 12-month follow-up (Mawhinney et al.^[58]^, 2017). D) NT SV harvesting prevents kinking of excessively long graft (Rueda et al.^[36]^, 2008).

## ENDOSCOPIC VEIN HARVESTING

Minimally invasive EVH of SV was introduced by Lumsden et al. in the same year as the NT technique^[64]^. This method of harvesting reduces leg wound complications such as infection, pain, and numbness, as well as improving cosmetic results. EVH was rapidly adopted and is now used in many centres worldwide and in the majority of CABG operations in the United States of America^[65]^. Whereas the extensive skin incisions used in open vein harvesting (OVH) allow removal with minimal surgical trauma to the conduit, there is a risk of higher local wound complications. When using either OVH or EVH, the SV should not be grasped with forceps, stretched, or over-distended since endothelial and other forms of vascular damage may affect graft patency. The benefits of EVH and improved wound healing are well accepted, nevertheless there are conflicting reports regarding the patency of SVGs prepared by this method. At best, it appears that the patency of EVH grafts is comparable to those prepared by OVH^[66-69]^. Although EVH is suggested to be ‘minimally invasive’ in terms of vessel exposure, wound healing, and scarring, manipulation by instruments and insufflation by CO_2_ cause considerable vascular trauma. This potentially impacts on the SV’s function as a bypass graft^[69-71]^. The damage inflicted effects not only on the endothelium, but also on other parts of the SV, including the intima and adventitia^[69,70]^, as well as the perivascular fat^[22]^, as seen in Figure 5 from Kiani et al., 2011^[69]^, and Kopjar et al., 2016^[22]^. When considering that EVH SVG’s patency is, at best, comparable to OVH’s patency, it seems reasonable to assume that NT SVGs will be superior to those prepared by EVH. This has been shown in a recent small, short-term study^[72]^. Interestingly, The National Institute for Health and Care Excellence (NICE) previous guidance in the United Kingdom advised that EVH should only be used with special arrangements^[73]^. This was based on data from the PREVENT IV trial where EVH grafts showed higher failure rates than OVH grafts. There was also a higher death rate and more myocardial infarction or revascularization in EVH *vs*. OVH grafts at three years postoperatively^[74]^. More recently, based on evidence published subsequently on large numbers of patients, NICE concluded that there was no increased occlusion rates or higher incidences of death, myocardial infarction, or reintervention for endoscopically harvested grafts^[75]^. However, there were comments regarding increased hospital costs for EVH and the importance of training and regular experience for any clinician doing this procedure (suggested to be up to 30 sessions or more). For more comprehensive reviews on EVH *vs*. NT SVG see Kopjar and Dashwood, 2016, and Kopjar et al., 2016^[22,76]^.

**Fig. 5:**
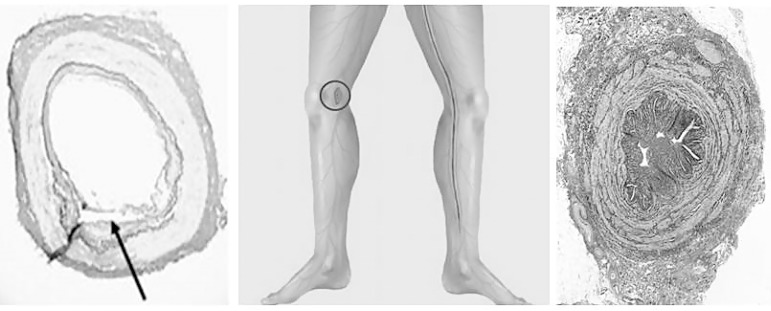
Saphenous vein (SV) histology of endoscopic vein harvesting (EVH) vs. no-touch (NT) techniques. Left panel is a transverse section of an SV prepared with EVH. The perivascular layers and endothelium are damaged. The arrow shows intimal tearing (Kiani et al.^[69]^, 2011). Middle panel to the left shows a diagram with a small incision above the knee used for insertion of EVH instruments. To the right, it shows a long incision in thigh and calf used for both open vein harvesting and NT harvesting. Right panel is a transverse section of a NT harvested SV with perivascular fat and adventitia intact and endothelium undamaged (Kopjar et al.^[22]^, 2016).

## RATIONALE

Better management and treatment undoubtedly improve long-term results in many aspects of life. This logic applies, among others, to classic cars, relationships, and to heart surgery. There is no doubt that the NT harvesting technique of SV is less traumatic than the C technique. With the available evidence, preservation of perivascular tissue on the NT SVG conserves normal vessel architecture, protects against distension-induced damage, and maintains endothelial NO, resulting in superior long-term results. Two ongoing multicenter trials, one in China (NCT03126409)^[77]^ and another in Sweden (NCT03501303) will shed further light on the role of NT vein grafts in CABG.

Reliable basic and clinical studies, in addition to logic and reason, have ultimately prevailed with the latest Class IIa recommendation of the NT technique in the 2018 ESC/EACTS guidelines on myocardial revascularization.

**Table:** 

Authors' roles & responsibilities	
NS	Substantial contributions to the conception or design of the work; or the acquisition, analysis, or interpretation of data for the work; drafting the work or revising it critically for important intellectual content; agreement to be accountable for all aspects of the work in ensuring that questions related to the accuracy or integrity of any part of the work are appropriately investigated and resolved; final approval of the version to be published
DS	Substantial contributions to the conception or design of the work; or the acquisition, analysis, or interpretation of data for the work; drafting the work or revising it critically for important intellectual content; agreement to be accountable for all aspects of the work in ensuring that questions related to the accuracy or integrity of any part of the work are appropriately investigated and resolved; final approval of the version to be published
BBP	Substantial contributions to the conception or design of the work; or the acquisition, analysis, or interpretation of data for the work; drafting the work or revising it critically for important intellectual content; agreement to be accountable for all aspects of the work in ensuring that questions related to the accuracy or integrity of any part of the work are appropriately investigated and resolved; final approval of the version to be published
TK	Substantial contributions to the conception or design of the work; or the acquisition, analysis, or interpretation of data for the work; drafting the work or revising it critically for important intellectual content; agreement to be accountable for all aspects of the work in ensuring that questions related to the accuracy or integrity of any part of the work are appropriately investigated and resolved; final approval of the version to be published
MD	Substantial contributions to the conception or design of the work; or the acquisition, analysis, or interpretation of data for the work; drafting the work or revising it critically for important intellectual content; agreement to be accountable for all aspects of the work in ensuring that questions related to the accuracy or integrity of any part of the work are appropriately investigated and resolved; final approval of the version to be published
